# Bioaugmentation of Native Fungi, an Efficient Strategy for the Bioremediation of an Aged Industrially Polluted Soil With Heavy Hydrocarbons

**DOI:** 10.3389/fmicb.2021.626436

**Published:** 2021-03-31

**Authors:** María Cecilia Medaura, Miriam Guivernau, X. Moreno-Ventas, Francesc X. Prenafeta-Boldú, Marc Viñas

**Affiliations:** ^1^Faculty of Engineering, National University of Cuyo, Mendoza, Argentina; ^2^GIRO Program, Institute of Agrifood Research and Technology (IRTA), Caldes de Montbui, Barcelona, Spain; ^3^Department of Sciences and Techniques in Water and Environment, University of Cantabria, Santander, Spain

**Keywords:** aged-polluted soil, high molecular weight polycyclic aromatic hydrocarbons, mycoremediation, native-fungal-bioaugmentation, fungal-bacterial interactions, indigenous hydrocarbonoclastic fungi

## Abstract

The concurrence of structurally complex petroleum-associated contaminants at relatively high concentrations, with diverse climatic conditions and textural soil characteristics, hinders conventional bioremediation processes. Recalcitrant compounds such as high molecular weight polycyclic aromatic hydrocarbons (HMW-PAHs) and heavy alkanes commonly remain after standard soil bioremediation at concentrations above regulatory limits. The present study assessed the potential of native fungal bioaugmentation as a strategy to promote the bioremediation of an aged industrially polluted soil enriched with heavy hydrocarbon fractions. Microcosms assays were performed by means of biostimulation and bioaugmentation, by inoculating a defined consortium of six potentially hydrocarbonoclastic fungi belonging to the genera *Penicillium*, *Ulocladium*, *Aspergillus*, and *Fusarium*, which were isolated previously from the polluted soil. The biodegradation performance of fungal bioaugmentation was compared with soil biostimulation (water and nutrient addition) and with untreated soil as a control. Fungal bioaugmentation resulted in a higher biodegradation of total petroleum hydrocarbons (TPH) and of HMW-PAHs than with biostimulation. TPH (C_14_-C_35_) decreased by a 39.90 ± 1.99% in bioaugmented microcosms vs. a 24.17 ± 1.31% in biostimulated microcosms. As for the effect of fungal bioaugmentation on HMW-PAHs, the 5-ringed benzo(a)fluoranthene and benzo(a)pyrene were reduced by a 36% and 46%, respectively, while the 6-ringed benzoperylene decreased by a 28%, after 120 days of treatment. Biostimulated microcosm exhibited a significantly lower reduction of 5- and 6-ringed PAHs (8% and 5% respectively). Higher TPH and HMW-PAHs biodegradation levels in bioaugmented microcosms were also associated to a significant decrease in acute ecotoxicity (EC_50_) by *Vibrio fischeri* bioluminiscence inhibition assays. Molecular profiling and counting of viable hydrocarbon-degrading bacteria from soil microcosms revealed that fungal bioaugmentation promoted the growth of autochthonous active hydrocarbon-degrading bacteria. The implementation of such an approach to enhance hydrocarbon biodegradation should be considered as a novel bioremediation strategy for the treatment of the most recalcitrant and highly genotoxic hydrocarbons in aged industrially polluted soils.

## Introduction

The bioremediation of soil polluted with petroleum hydrocarbon has many advantages compared to physicochemical techniques, but it also presents challenges due to the heterogeneity and variable concentration of contaminants, as well as the diverse site environmental conditions (Atlas and Philip, 2005; Singh, 2006). The tendency of heavy hydrocarbon fractions to adhere onto the soil organic and mineral particles, a process dubbed as pollution aging, reduces their bioavailability to microorganisms and, consequently, hinders the overall biodegradation efficiency (Hatzinger and Alexander, 1995). As a result, biodegradation rates may slow down and end-point concentrations of heavy petroleum fractions might stabilize at values that represent an unacceptable risk to the environment (Nocentini et al., 2000). This scenario is quite common in soils with a high initial concentration of hydrocarbons (Viñas et al., 2005a; Ren et al., 2018). In these cases, compounds that are recalcitrant to biodegradation, such as polycyclic aromatic hydrocarbons (PAHs) of high molecular weight (HMW, which have more than three benzene rings), heavy alkanes (the saturated aliphatic fraction with carbon chain-lengths that range C_30_-C_40_), and the unresolved fraction (complex mixtures of saturated hydrocarbons), might still be present at concentrations above regulatory limit values defined in many countries. Poor biodegradation of hydrocarbon pollutant in soil conditions is often the result of limited bioavailability and non-optimal environmental conditions, rather than because of the lack of biodegrading microflora (Manilal and Alexander, 1991). Therefore, many investigations have focused on the microorganisms that have an ability to biodegrade residual hydrocarbons in soil for cleaning up contaminated sites under growth-limiting conditions (Dhawale et al., 1992; Boonchan et al., 2000; Zheng and Obbard, 2002). Previous studies have shown the advantages of fungi over bacteria for the biodegradation of HMW-hydrocarbons in contaminated soils (Aranda et al., 2017; Prenafeta-Boldú et al., 2019): (i) secretion of several low substrate specificity enzymes (e.g., laccases, lignin peroxidases, and Mn peroxidases) (Harms et al., 2011); (ii) osmo- and xerotolerance of several fungal species that confers an ability to grow in rather extreme and fluctuating environments (Worrich et al., 2017; González-Abradelo et al., 2019; Peidro-Guzmán et al., 2020); and (iii) the capacity of filamentous fungi to form mycelial networks that are often hydrophobic and that might cover several hectares of soil, enhancing the access to hydrocarbon contaminants (Wick et al., 2007; Furuno et al., 2012; Bielèik et al., 2019). These abilities are of particular interest in case of the less water soluble HMW-PAHs, which are strongly adsorbed onto the organic matter and are therefore less available for microbial metabolism (Ghosal et al., 2016).

Previous studies have focused on the capacity of white rot fungi (WRF), generally belonging to the basidomycetes, to unspecifically degrade PAHs through extracellular lignin-modifying enzymes (Tortella et al., 2005; Cerniglia and Sutherland, 2010). However, these abilities might be limited in polluted soils that are devoid of lignocellulosic matter and/or humidity, reducing their competitiveness against the resident microbiota (Marco-Urrea et al., 2015). Other fungal groups, mainly ascomycetes belonging to the classes *Sordariomycetes* (i.e., *Fusarium*) and *Eurotiomycetes* (i.e., *Aspergillus* and *Penicillium*), and the former phylum of the zygomycetes, are found ubiquitously—also in polluted soils—, and can metabolize a broad spectrum of organic compounds, such as sugars, cellulose, starch, proteins and lipids, as well as hydrocarbons, including PAHs, by the action of intracellular P450 cytochrome monooxygenases (Aranda et al., 2017). Previous bioaugmentation studies selected autochthonous saprophytic ascomycetes from soils with a prolonged pollution history, assuming that they are more likely to survive and metabolize PAHs than organisms introduced from elsewhere (Atagana et al., 2006; D’Annibale et al., 2006; Mancera-López et al., 2008; Fayeulle et al., 2019). However, hydrocarbon biodegradation in fungi is of co-metabolic nature in most cases and requires the concurrence of an additional carbon and energy source. Hence, other microbial populations are needed to further biodegrade the PAH partly oxidized intermediates. Furthermore, bioaugmentation with allochthonous fungi has been linked to both an important increase of heterotrophic bacteria and of pollutant degradation (D’Annibale et al., 2006; Federici et al., 2007; Ma et al., 2015). These results pointed to the relevance of the fungal-bacterial interactions, and to the potential of fungal inoculants as bioremediation enhancers in polluted soils.

The biochemical mechanisms of extracellular lignin modifying enzymes (i.e., laccase, manganese peroxidase and lignin peroxidase) produced by lignin-degrading basidiomycetes have commonly been described as the prevailing metabolic routes for PAH biodegradation in fungi (Reddy, 1995; Kües, 2015). However, in PAH polluted soil under field conditions, lignin modifying enzymes are expressed poorly due to the scarcity of lignocellulosic substrates, so that these fungi might be outcompeted by other specialized hydrocarbon-degrading microorganisms with alternative pathways, such as ring hydroxylating oxygenases in bacteria, and cytochrome P450 monooxygenases (CYPs) that have been deeply assessed in several Ascomycota for PAHs biodegradation (Marco-Urrea et al., 2015). In fact, CYP enzymes play pivotal roles in fungal metabolism, encompassing housekeeping reactions, detoxification of chemicals, and adaptation to environmental conditions (Durairaj et al., 2016). Among Ascomycota fungi, CYP-mediated hydrocarbon biodegradation is the prevalent mechanism described in species that belong to the genera *Fusarium*, *Aspergillus*, and *Penicillium* (Aranda, 2016; Loss et al., 2019). The fungal CYP biodegradation pathway of PAHs encompasses two main phases. First, hydrocarbons are subjected to an initial oxidation by CYPs and epoxide hydrolases, which transform PAHs to hydroxy, dihydroxy, dihydrodiol, and quinone derivatives, and by the subsequent action of transferases and quinone reductases (Aranda et al., 2017). Then, the oxidized metabolites are conjugated with other molecules and stored in organelles and lipid-vesicles, inside the fungal cell as previously described in *Fusarium solani* (Verdin et al., 2005; Fayeulle et al., 2014), or secreted in a more soluble and biodegradable form (Meulenberg et al., 1997; Capotorti et al., 2005). Regarding aerobic PAHs biodegradation pathways in bacteria, they are characterized by an initial oxidation of the aromatic ring *via* the incorporation of molecular oxygen by dioxygenase enzymes to form *cis*-dihydrodiols-based metabolites that are further converted to diol-dihydroxylated compounds, such as catechol intermediates that are then cleaved by dioxygenases to generate products that enter the TCA cycle (Kanaly and Harayama, 2000; Peng et al., 2008; Vila et al., 2015). Therefore, a complex mixture of metabolites associated to different catabolic pathways from fungi and bacteria, may form in biostimulated and bioaugmented soils, and could lead to different soil ecotoxicity scenarios.

The objective of this study was to test the extent to which an autochthonous fungal inoculum can promote a greater biodegradation of high molecular weight hydrocarbons, mainly 5–6 ring PAHs, in an industrially aged oil-polluted soil, and to identify the predominant bacterial populations that can be biostimulated by the activity of the bioaugmented fungi, in comparison to a more conventional biostimulation approach (nutrient and water supplementation), and in contrast to untreated soil as a control. The assessments were performed in microcosms and an array of culture-dependent methods, ecotoxicological assays, and molecular profiling techniques were implemented for the isolation, cultivation, and characterization of complex microbial populations and biochemical parameters.

## Materials and Methods

### Soil Material

Soil samples were taken in Mendoza, Argentina, from a deposit of soils previously treated by landfarming for 1 year and kept in outdoor piles during 10 years under extreme climatic conditions (i.e., dry arid climate, average rainfall below 200 mm, with almost 3,000 h of sunshine throughout the year, summer average temperature of 28 and 7°C in winter, with daily temperature fluctuations of +12 and +42°C in summer and 2 and 15°C in winter). The soil was air dried and sieved with a 6 mm grid previous to microcosm experiments and 2 mm grid for characterization analysis. Soil samples were stored aerobically at 4°C in the dark until use.

### Analytical Methods for Soil Characterization

The soil samples were characterized according to the following standard physicochemical parameters and methodologies (APHA et al., 2005): texture; pH; electric conductivity; total nitrogen; available phosphorous and moisture. Elemental analysis of C (carbon), H (hydrogen), N (nitrogen), and S (sulfur) was performed with a LECO Truspec CHNS (LECO Corporation, United States). Based on the chemical characteristics of the soil hydrocarbon contamination, the TNRCC Method 1005 (Texas Natural Resource Commission, 2001) was used for extraction and quantitative analysis of Total Petroleum Hydrocarbons (TPH), as well as for the evaluation of the relative distribution of each TPH in the sample extracts. This method used n-pentane (chromatographic grade, Sintorgan, Buenos Aires, Argentina) as solvent, which was then analyzed by gas chromatography/flame ionization detection (GC/FID) for identifying and quantifying hydrocarbons between nC6 and nC35 by comparing chromatographic profiles with those from reference n-alkane references (C8-C40 standard kit, AccuStandard Inc., New Haven, CT, United States). PAHs were identified and quantified by gas chromatography/mass spectrometry (GC/MS) under the selected ion monitoring mode (SIM), by measuring m/z area signal of 16 EPA priority PAHs according to the EPA Method 8270D. Before the n-pentane extraction, 20 μg g^–1^ of o-terphenyl and α-androstene (AccuStandar Inc., New Haven, CT, United States) were added in an acetone solution (1 mg mL^–1^) to each sample as surrogate internal standards. Acetone was allowed to evaporate and 10 g of soil were suspended in 10 mL of n-pentane in a sealed screw PTFE cap glass vial, shaken by vortex during 5 min and allowed to settle overnight. Thereafter, 2 mL of sample extracts were transferred to auto sampler vials for further chromatographic analysis.

The biodegradation of saturated compounds (alkanes) was verified using a Clarus 500 Perkin Elmer GC/FID. Compounds were separated on a HP-5 capillary column [25 m by 0.32 mm (i.d.), 0.25-μm film thickness (Hewlett-Packard)]. Column temperature was held at 42°C for 5 min and then programmed to reach 300°C at a rate of 10°C min^–1^. This final temperature was held for 80 min. Detector and inlet temperatures were set at 320 and 300°C, respectively. The helium flow was 1.1 mL min^–1^ and the injection volume was 1 μL. Concentration ranges of aliphatic hydrocarbons were calculated from the total areas of the peaks in the chromatograms corrected with internal standards areas and standards of aliphatic hydrocarbons (C12-C40 range) of known concentration.

A HP-5 capillary column [30 m by 0.32 mm (i.d.) Hewlett-Packard] with 0.25-μm film thickness and helium as a carrier gas (10 psi) were used. Column temperature was held at 50°C for 5 min and then programmed to 250°C at a rate of 10°C min^–1^. This final temperature was held for 35 min. Injector, transfer line and analyzer temperatures were set at 320°C. Injection was in splitless mode keeping the split valve closed for 30 s. The targets for this analysis were the 16 priority EPA PAHs. The obtained reconstructed ion chromatograms were numerically compared with internal standards (16 EPA PAHs standard, AccuStandar Inc., New Haven, CT, United States) for the biodegradation estimation of each analyte in soil samples.

The total TPH concentrations estimated according to the EPA Method 418.1, referred hereafter as TPH-IR, were considered as kinetic biodegradation parameter.

### Isolation of Autochthonous Fungi

Dry soil (10 g) was added to 100 mL of a sterile NaCl 0.09% (w/v) solution in a 200 mL Erlenmeyer flask and shaken for 20 min on a shaker. After solid particles were allowed to settle for 30 min, serial dilutions were prepared and 100 μL of each dilution were spread homogeneously on the surface of three replicate malt yeast extract agar plates (MYEA: 2% malt extract, 0.2% yeast extract, 1.5% agar, pH 7.0) and on mineral agar amended with phenantrene, as standard PAH, (20 mg PHE crystals L^–1^, PHEN medium) as the sole carbon and energy source [PHEN composition per L: 4.5 g KH_2_PO_4_; 0.5 g K_2_HPO_4_; 2.0 g NH_4_C; 0.1 mg MgSO⋅7H_2_O; 120 mg FeCl_3_; 50 mg H_3_BO_3_; 10 mg CuSO_4_⋅5H_2_O; 10 mg KI; 45 mg MnSO_4_⋅H_2_O; 20 mg Na_2_MoO_4_⋅H_2_O; 75 mg ZnSO_4_⋅H_2_O; 50 mg CoCl_2_⋅6H_2_O; 20 mg AlK(SO_4_)_2_⋅12H_2_O; 13.25 g CaCl_2_⋅H_2_O; 10 g NaCl]. PHEN agar was also supplemented with a solution of sterilized chloramphenicol (0.02%) and was autoclaved at 120°C for 20 min before dispensing into Petri dishes. PHEN was selected according to previous studies being the most common PAH source to distinguish viable PAH-degrading bacteria and fungi on agar plates (Viñas et al., 2005b; Hesham et al., 2016; Agrawal et al., 2018) and in base of previous analyses of the industrial soil where it was one of the PAH with the highest concentration and prevalence.

Inoculated agar plates were incubated at 25°C during 15 days (MYEA) and 21 days (PHEN) days sheltered from light. The observation of morphological characteristics of several colonies (hyphal tips, mycelium shape, color, surface, elevation, form, and grown velocity) that appeared to belong to different representative isolates were transferred to new MYEA plates.

Colonies were subcultured in MYEA until pure cultures were obtained. Twelve strains were morphologically described as being different on MYEA slants and only one of them was able to grow on PHEN agar. Strains grown only on MYEA agar were labeled correlatively from A1 to A11, and B1 corresponded to the one growing on PHEN agar. All isolates were maintained on MYEA slants incubated at 20°C.

### Preparation of Fungal Inoculum

The inoculum consisted of a suspension of mycelia from a defined fungal consortium of twelve selected fungal isolates, prepared as described by Potin et al. (2004). Every one-week-old MYEA and PHEN Petri dish cultures of the twelve isolates was washed with 4 mL of sterile deionized water. Mycelium fragments were removed from the spore suspension by filtration through sterile glass wool. 100 μL of every spore suspension were mixed to form a single one which was estimated using a Thoma chamber. Before inoculation into de soil, the spore suspension was mixed with a solution of glucose and sucrose, each at 5 mg g^–1^ soil, as carbon source and kept in agitation for 36 h at 25°C in order to induce spore germination and hyphal elongation. The germinated spores were microscopically controlled and added to the soil in calculated volumes to give an equal contribution of each strain (1:12) and a final total spore concentration of 10^4^ spores g soil^–1^. None lignocellulosic amendment was added to the soil.

### Microcosms Assays

All the microcosm treatments were conducted in triplicate with 1,200 g of 6 mm sieved soil inside 5 L glass recipients with plastic lids and was incubated for 120 days at room temperature. Three different microcosm treatments were designed: (i) bioaugmentation (B), consisting of soil inoculated with fungi up to an initial concentration of 10^4^ spores per gram of soil. Soil moisture was maintained regularly at 20% w/w by gravimetry and K_2_NO_3_ and K_2_HPO_4_ were added on days 30 and 90 to prevent the C:N:P ratio descended below 100:10:1; (ii) biostimulation (BS), in which soil moisture was maintained regularly at 20% w/w by gravimetry, and K_2_NO_3_ and K_2_HPO_4_ were added on days 30 and 90 to prevent the C:N:P ratio descend below 100:10:1, the optimal for bacterial biostimulation (Bossert and Bartha, 1984; Morgan and Watkinson, 1989; Mills and Frankenberger, 1994; Chaineau et al., 2005); and (iii) a control (C) formed by air-dried untreated contaminated soil (13% humidity), with the aim to evaluate abiotic losses of hydrocarbons in the absence of enough water content. In each microcosm, air renewal occurred weekly through manual removal by using cleaned and sterilized stainless-steel spatulas under sterile flow cabinet. As microcosm B includes biostimulation by the addition of nutrients plus bioaugmentation, BS also represented a control treatment for B. Soil samples from each microcosm (120 g) were collected manually with clean and sterilized (ethanol 70%) stainless steel spatulas on days 0, 30, 60, and 120 for microbiological and physicochemical analysis.

### Ecotoxicological Characterization

Acute hydrocarbon toxicity in soil microcosms was assessed on soil samples collected on days 0, 30, 60, and 120 by quantifying the EC50 with the Microtox^TM^ Basic Solid-Phase Test (Microtox BSPT, AZUR Environmental, CA, United States). This *Vibrio fischeri based* bioluminiscence inhibition assay has been previously described as a versatile and suitable test to detect polar compounds that could be accumulated in soil during biodegradation processes (Parvez et al., 2006; Sabaté et al., 2006). Soil samples were diluted at an initial and final concentration of 200 and 0.781 g soil L^–1^, respectively, and were osmotically corrected. Cells were resuspended with Microtox resuspension agent and analyzed spectrophotometrically according to the instructions of the manufacturer. Statistical regressions between Microtox readings and TPH concentrations were obtained by log-log plots using the Statistical software (IBM, United States). The resulting EC50 values were defined as the antilog of the regression line‘s intersection point.

### Quantification of Microbial Populations in Soil

Soil microbial counts were performed using a miniaturized most probable number (MPN) method in 96-well microtiter plates, with eight replicate wells per dilution (Wrenn and Venosa, 1996). Total heterotrophs were counted in tryptone soy broth and aromatic hydrocarbon-degraders were counted in liquid mineral medium (BMTM) containing a mixture of phenanthrene (0.5 g L^–1^), with fluorene, anthracene, and dibenzothiophene (each at a final concentration of 0.05 g L^–1^) according to Viñas et al. (2005b).

The PAHs were administered in a pentane solution that was allowed to evaporate in each well, where the BMTM mineral medium was subsequently added. Aged soil was used as the starting point (day 0). MPN plates were incubated at room temperature (25 ± 2°C) for 30 days. Positive grown wells were detected by turbidity (heterotrophs) and by the presence of coloration (brownish/yellow) for PAH degraders. Microbial population in soil microcosms were quantified on days 0, and after 30, 60, and 120 days of incubation. Since no antifungal agent was added to the medium, the total heterotrophs quantification includes both bacteria and fungi.

### Screening of Ligninolytic Enzymes

Pure strains of previously isolated fungi were cultivated on mineral agar amended with ABTS [2,2′-azine-bis (3-etilbenztiazoline-6-sulfonic acid)], tannic acid, and Red Orange (RO) to screen their laccase, phenol oxidase, and manganese peroxidase secretion. These chromogenic agar methods were characterized by developing a pigmentation in the oxidized state that was green for ABTS, dark orange for tannic acid, and an orange red halo for RO. The culture mineral medium was prepared as described by Prenafeta-Boldú et al. (2001). Per liter of dematerialized water: 4.5 g KH_2_PO_4_; 0.5 g K_2_HPO_4_; 2.0 g NH_4_C; 0.1 g MgSO_4_⋅7H_2_0; 120 mg FeCl_3_; 50 mg H_3_B0_3_; 10 mg CuSO_4_⋅5H_2_O; 10 mg KI; 45 mg MnSO_4_⋅H_2_O; 20 mg Na_2_MoO_4_⋅H_2_O; 75 mg ZnSO_4_⋅H_2_O; 50 mg CoCl_2_⋅6H_2_O; 20 mg AlK(SO_4_)⋅12H_2_O; 13.25 g CaCl_2_⋅H_2_O; 10 g NaCl; 2 g Glucose; 100 mg nicotinic acid; 200 mg calcium pantothenate; 25 mg cyanocobalamin; 100 mg inositol; 20 mg p-amino benzoate; 50 mg thiamin⋅HCl; 25 mg pyridoxin⋅HCl; 10 mg biotin; 10 mg riboflavin; 10 mg folic acid;10 mg thioctic acid and 16 g agar). This medium was supplemented with ABTS (500 mg), tannic acid or orange red chromogenic substrate (100 mg each) according to Pointing (1999). A collection strain of *Trametes versicolor* (ATCC 20869^TM^ ) was used as a positive control for ligninolytic activity (laccase, Mn peroxidase and polyphenol oxidase).

### Molecular Methods

A 250 mg sample of biomass from each fungal isolate and of soil from each microcosm was placed in a sterile tube and stored at −20°C prior to analysis. DNA was extracted by a bead beating protocol using the PowerSoil^TM^ DNA extraction kit (MoBio Laboratories, Inc., Carlsbad, CA, United States), following the manufacturer’s instructions. A further purification of soil DNA extracts was needed using the Clean DNA Wizard Kit (Promega, WI, United States) to avoid PCR inhibition.

Total genomic DNA from the original soil and from microcosm test samples were characterized by PCR-DGGE (Polymerase Chain Reaction and Denaturing Gradient Gel Electrophoresis) using specific primers for amplifying the hypervariable V3–V5 region of the bacterial 16S rRNA gene (F341GC/R907: Yu and Morrison, 2004). For the identification of isolated fungal strains, the ITS1-ITS2 rRNA region was amplified, which also contains the 5.8S coding region (ITS1F-ITS4R Esteve-Zarzoso et al., 1999). All the PCR reactions were performed with a Mastercycler (Eppendorff, Hamburg, Germany) and each reaction mix (25 μL mix/reaction) contained 1.25 U of Ex Taq DNA polymerase (Takara Bio, Otsu, Shiga, Japan), 12.5 mM dNTPs, 0.25 μM of each primer and 100 ng of DNA. The obtained amplicons from the 16S rRNA were loaded in an 8% (w/v) polyacrylamide gel with a chemical denaturing gradient ranging from 30 to 70% [100% denaturant contains 7 M urea and 40% formamide (w/v)], and electrophoretically resolved in a DGGE-4001 equipment (CBS Scientific Company, Del Mar, CA, United States). The electrophoresis was carried out at 60°C and at 100 V for 16 h in a 1X TAE buffer solution (40 mM Tris, 20 mM sodium acetate, 1 mM EDTA, pH 7.4).

The DGGE gel was stained for 45 min in 1 × TAE buffer solution containing SybrGold^TM^ (Molecular Probes, Inc., Eugene, OR, United States) and then scanned under blue light by means of a blue converter plate (UV Products Ltd., Cambridge, United Kingdom). Predominant bacterial DGGE bands were excised with a sterile filter tip, resuspended in 50 μL sterilized Milli-Q water and stored at 4°C overnight. A 1:50 dilution of the supernatant was subsequently reamplified by PCR as described previously and sequenced by using the R907 primer.

Sanger sequencing of amplicons of isolated fungal strains were performed with the ITS4 primer. Sequencing was accomplished using the ABI Prism Big Dye Terminator Cycle-Sequencing Reaction Kit v. 3.1 (Perkin–Elmer Applied Biosystems, Waltham, MA, United States) and an ABI 3700 DNA sequencer (Perkin–Elmer Applied Biosystems, Waltham, MA, United States), according to the manufacturer’s instructions. Sequences were edited using the BioEdit software package v. 7.0.9 (Ibis Biosciences, Carlsbad, CA, United States). The sequences were aligned with the NCBI genomic database using the BLAST search alignment tool (Altschul et al., 1990) and were related to the phylogenetic groups using the RDP Naive Bayesian Classifier (Wang et al., 2007). The 16S rRNA bacterial and ITS1 rRNA fungal gene nucleotide sequences determined in this study were deposited into the Genbank database under accession numbers F779671-JF779675 and JF729186-JF729196.

### Statistical Analysis

The normal distribution of the data was determined with the Kolmogorov-Smirnov test and the statistically significance of differences between treatments were evaluated by one-way analysis of variance (ANOVA). The homoscedasticity between the groups was studied using the Levene test. The *post hoc* tests were carried out using Tukey, when there was homoscedasticity, and by using Games Howel when there was none. In the case of non-normal distributions, the Kruskal–Wallis test was used to determine whether or not the consequences of treatment were significant. ANOVA and *post hoc* analysis with Tukey’s multiple-range test with a significance level of 0.05, was applied to the results to determine their statistical significance, (*P* < 0.05) by the use of SSPS V15, IMB SPSS Statistics, United States. DGGE bands of the samples were processed by GeneTools analysis software (Syngene, United Kingdom) and they were studied by multivariate detrended correspondence analysis (DCA) by using the Canoco Software (version 5, Microcomputer Power, NY, United States).

## Results and Discussion

### Soil Characterization

The soil was loamy clay, neutral (pH: 7.6), with a high electric conductivity (1,416 μS cm^–1^) and a low content of heavy metals. Chemical analysis showed a total nitrogen and phosphorous content of 300 mg-N kg^–1^ and 6 mg-P kg^–1^, respectively, a moisture of 12% (w/w), and a water holding capacity 34% (w/w). Elemental analysis yielded a molar ratio C(carbon):H(hydrogen):N(nitrogen):S(sulfur) of 13:3:0.2:0.6, which indicated that the C:N ratio was slightly above the recommended values (25:1:1 to 38:1:1), according to Atagana (2009) and Alexander (1999), for the fungal PAHs bioremediation under an optimum water and oxygen content. The comparative elemental analysis of a pristine soil sample taken near the polluted site under study indicated that the carbon content in the later was increased from 2% up to 13% (Supplementary Table 1). Likewise, the total nitrogen content in the contaminated soil was fivefold higher with respect to the non-polluted soil. Such high values might be explained by the fertilization applied during the previous landfarming activities. Further increases in nitrogen content in bioaugmented and biostimulated microcosms (BS), in relation to the control, were due to the addition of inorganic nutrients.

The content of aliphatic and aromatic hydrocarbon fractions evidenced that readily degradable compounds had already been removed from soil during previous landfarming treatments in the polluted site. Concentrations of all PAHs subjected to regulation exceed the threshold values proposed by the Argentinian and European legislation for agricultural and residential soils (Table 1). Heterotrophic and hydrocarbonoclastic microbial populations quantified by the MPN were relatively high (heterotrophs: 2 × 10^7^ MPN g soil^–1^, alkanes and PAHs degraders: 8 × 10^5^ and 1 × 10^4^ MPN g soil^–1^ respectively), indicating that there was an abundant indigenous microbial population with hydrocarbon-degrading capabilities.

**TABLE 1:** Aliphatic and aromatic hydrocarbon composition of the initial contaminated soil.

Aliphatics			PAHs		
N° of C	Content (mg Kg^–1^)^*a*^		N° of rings	Mean (mg Kg^–1^)^*a*^	Threshold^*b*^ (mg Kg^–1^)
C10-C11	1.00 (±)2.22	Naphtalene (NA)	2	N.D.^*e*^	0.10–5.00 – 50.00
C12-C13	31.00(±)1.94	Acenaphtylene (ACY)	3	N.D.^*e*^	N.S.^*f*^
C14-C15	654.00(±)32.00	Acenaphtene (ACE)	3	N.D.^*e*^	N.S.^*f*^
C16-C17	2,462.00(±)83.23	Fluorene (FLO)	3	N.D.^*e*^	N.S.^*f*^
C18-C19	1,986.00(±)51.80	Phenanthrene (PHE)	3	9.00(±)0.00	0.10–5.00 – 50.00
C20-C21	1,473.00(±)58.60	Anthracene (ANT)	3	N.D.^*e*^	0.10–5.00 – 50.00
C22-C23	912.00(±)18.67	Fluoranthene (FLT)	4	N.D.^*e*^	N.S.^*f*^
C24-C25	906.00(±)12.60	Pyrene (PYR)	4	10.41(±)1.46	0.10–5.00 – 50.00
C26-C27	1,396.00(±)94.16	Benzo(a) anthracene (BAA)	4	7.36(±)0.76	0.10–1.00 – 10.00
C28-C29	1,797.00(±)30.92	Chrysene (CHR)	4	14.22(±)0.76	N.S.^*f*^
C30-C31	1,571.00(±)21.07	Benzo(b,k)fluoranthene (BF)	5	6.45(±)0.68^*c*^	0.10–1.00 – 10.00
C32-C33	3,488.00(±)63.70	Benzo (a)pyrene (BAP)	5	17.50(±)0.42	0.10–1.00 – 10.00
C34-C35	225.00(±)41.50	Dibenz(a,h)anthracene (DBA)	5	N.D.^*e*^	0.10–1.00 – 10.00
		Indeno(1,2,3-cd)pyrene (IND)	6	N.D.^*e*^	0.10–1.00 – 10.00
		Benzo(g,h,i)perylene (BPL)	6	23.23(±)0.22	0.10–1.00 – 10.00
Total Alkanes	2,060.00(±)104.35^*d*^		Total 3,4-rings PAHs	40.99	
TPH C10-C35	16,114.00(±)1,936		Total 5,6-rings PAHs	47.18	
**TPH (IR) TPH threshold^*b*^**	**45,000k 10,000**		**Total resolved PAHs**	**88,17**	

*^a^Means and standard deviations of three replicates; soil dry weight basis.*

*^b^Threshold Argentinian values for domestic-agricultural-industrial use soil respectively.*

*^c^Benzo(b)fluoranthene and Benzo(k)fluoranthene are detected in the same peak.*

*^d^Sum of resolved alkanes.*

*^e^Non detectable: concentration under Detection limit: 0.01 mg Kg^–1^.*

*^f^Not specified: concentration not specified by Argentinian legislation.*

### Isolation and Molecular Identification of Fungi

Twelve morphologically different fungal strains were isolated successfully from the aged polluted soil and were maintained as pure cultures in MYA slants. Only one of these strains, labeled as B1, was able to grow on PHEN agar, while the remaining were labeled correlatively from A1 to A11. The identification of these fungi was attempted by sequencing the ITS1 rRNA region. Only five of the 12 isolated fungal strains turned out to be different after sequencing (Table 2). All sequences showed high similarity (99–100%) with other sequences from type material deposited in GenBank. Sequence matches from the isolated fungi corresponded to *Penicillium* spp., *Penicillium crysogenum, Ulocladium* spp., *Ulocladium atrum, Aspergillus terreus* (three strains), *Fusarium oxysporum* (four strains), and *Aspergillus parasiticus* (three strains). These fungi are rather fast-growing and highly sporulating ascomycetes and might, therefore, not be representative of the dominant or active fungal hydrocarbonoclastic diversity of the soil. Nevertheless, all these species have commonly been described as HMW-PAHs degraders (D’Annibale et al., 2006; Cerniglia and Sutherland, 2010; Romero et al., 2010). Of particular interest is the genus *Fusarium* in that several strains from this group have been associated to the biodegradation of petroleum hydrocarbons, as reviewed recently by Prenafeta-Boldú et al. (2019). This extremely diverse and ubiquitous genus is widely distributed in soils but also in plant material, either as harmless saprobe or as a pathogen. The assimilation of benzo(a)pyrene as the sole source of carbon and energy by a *F. solani* strain has been demonstrated under laboratory conditions (Rafin et al., 2000). The involvement of CYPs, lignin peroxidases, and laccase enzymes was suggested as fundamental for the biodegradation process in this fungus (Rafin et al., 2008). Furthermore, the translocation of the 4-ringed pyrene along mycelial networks of the closely related *Fusarium oxysporum* has also been demonstrated (Guivernau et al., 2012). The relatively high frequency of isolation of *Fusarium* strains in this work could be an indication of the predilection of this genus toward PAHs polluted environments.

**TABLE 2:** Preliminary identification of the isolated fungal strains by sequencing of the ITS1-ITS2 rRNA region, and enzymatic screening of the ligninolytic activity.

Isolated fungal strain	Accession number	Closest match in GenBank (accession no.) Closest type strain in GenBank (accession no.)	Similarity^a^ (%)	Phylogenetic group^b^	Enzymatic activity^c^		
	
					Laccase	Polyphenol Oxidase	Mn Peroxidase
MYEA 1	JF779671	*Penicillium* sp. BM (GU566211) *Penicillium chrysogenum* strain ZJ-T2 (HQ882177)	100 99	*Ascomycota/Eurotiales*	−	−	−
MYEA 2	JF779672	*Ulocladium* sp. N4 (GQ169445) *Ulocladium atrum* strain UMAH (HM101093)	100 99	*Ascomycota/Pleosporales*	+	−	±
MYEA 3,7,9	JF779673	*Aspergillus terreus* isolate UOA/HCPF (GQ461911)	99	*Ascomycota/Eurotiales*	−	−	−
MYEA 5,6,8 PHE-MYEA 12	JF779674	*Fusarium oxysporum* strain BD (GU566205)	100	*Ascomycota/Hypocreales*	+	−	±
MYEA 4,10,11	JF779675	*Uncultured Trichocomaceae* clone 3B4 (FN689694) *Aspergillus parasiticus* isolate ASPpar1 (GQ131879)	100 99	*Ascomycota/Eurotiales*	−	−	−
Control(+) *Trametes versicolor*					+	+	+

*^a^Sequences were matched with the closest relative from the GenBank database.*

*^b^Sequences were matched with the closest relative from the GenBank database. Phylum/Order is represented.*

*^c^Enzyme activities are expressed as (+), (−) or (±) according to the intensity of the color developed in the corresponding medium: laccase (ABTS and Caffeic acid), polyphenol oxidase (Tannic acid), and Mn peroxidase (Red Orange).*

### Screening of Ligninolytic Enzyme Production

Five out of the twelve isolates were positive for the appearance of a dark halos around fungal colonies in mineral ABTS agar, which is an indication of extracellular laccase secretion. These positive strains included *Fusarium oxysporum* (strains A5, A6, A8, and B1), which displayed dark-green halos, and *Ulocladium* sp. (strain A2) with a dark-purple halo (Supplementary Figure 1A). This later strain A2 was the only one that also displayed a halo in tannic acid agar (Supplementary Figure 1B), pointing to the secretion of polyphenol oxidases, whereas all the other tested strains were negative (Table 2). No indication of Mn peroxidase secretion was observed through coloration of RO agar in any of the tested strains, with the exception of the control ligninolytic fungus *Trametes versicolor*, which tested positive on all chromogenic agar assays.

Despite limited knowledge at the proteomic level, extracellular laccases from *Fusarium oxysporum* have been proposed as one of the main factors responsible for its virulence as a plant pathogen. This claim has been substantiated by the finding of three genes that may encode laccases *sensu stricto* in a comparative genomic study of twelve *F. oxysporum* strains (Kwiatos et al., 2015). Putative laccase and phenol oxidase production observed in colonies of the darkly pigmented *Ulocladium* sp. (class Dothideomycetes) is also noteworthy. These two enzymes are involved in the fungal melanization process and laccases have also been found to be secreted by other dematiaceous fungi from the Dothydeomycetes (Tetsch et al., 2005). Fungal laccases have been recognized as industrial enzymes that might also be of interest in the mycoremediation of PAH-polluted soils. For example, Li et al. (2018) revealed that the white-rot fungi *Pycnoporus sanguineus* transformed phenanthrene *via* laccase and CYP, and benz[a]anthracene *via* laccase. Despite the fact that further research is needed, our enzymatic screening provides a first hint toward the involvement of specific enzyme systems in the fungal biodegradation of PAHs. In fact, a previous study in aged-creosote polluted soils by means of 16S rRNA amplicon sequencing, revealed that the main fungal community in a biostimulated soil was dominated by native ascomycetes (i.e., *Fusarium* spp. and *Scedosporium* spp.) before and after the biostimulation process. These species were even dominant after bioaugmentation with ligninolytic strains—and supplementation with lignocellulosic material—of the WRF *Trametes versicolor* and *Lentinus tigrinus* (Lladó et al., 2015). Further research must also consider other fungal enzymes, such as unspecific peroxygenases (UPOs), that act as the extracellular counterparts of intracellular CYP systems, and might contribute to the biodegradation of PAHs as well (Karich et al., 2017).

### Biodegradation of Petroleum Hydrocarbons in Soil Microcosms

#### Total Petroleum Hydrocarbons and Alkane Fractions

**FIGURE 1:**
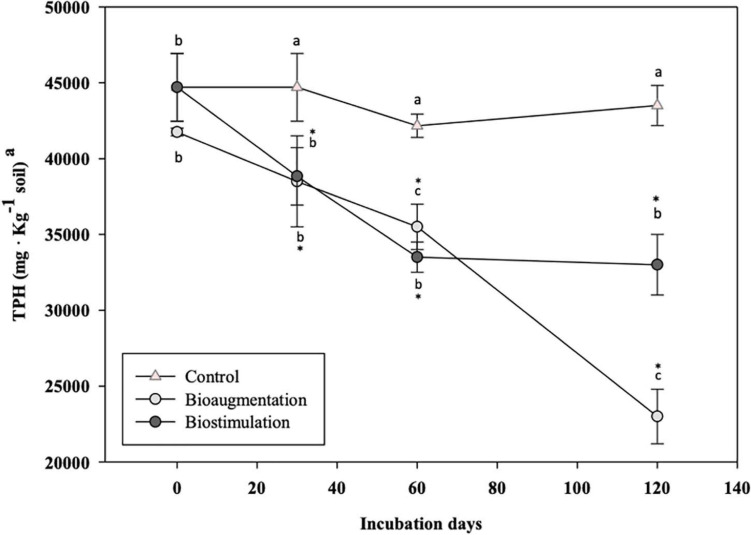
TPH concentration evolution in microcosm assays along 120 days of incubation.^a^Data are the means of three independent experiments. ^a,b,c^Same lower-case letters indicate lack of statistically significant difference (*P* < 0.05) between each biostimulation or bioaugmentation treatment. *Asterisk represents the occurrence of significant differences between B and BS treatments with control C (*P* < 0.05). Values are expressed in terms of dry weight.

Total petroleum hydrocarbons decreased by a 39.90 ± 1.99% in fungal-bioaugmented microcosm assays (B) after 120 days of incubation, in contrast to a 24.17 ± 1.31% in BS, and just a 2.69 ± 0.13% in the control (C) (Figure 1). After testing for normality (Kolmogorov–Smirnov test), a one-way ANOVA comparing treatments B and BS with the control C, and B with BS, confirmed that the observed differences were statistically significant (*P* = 0.004 and *P* = 0.007, respectively). The differences between treatments B and BS demonstrates that bioaugmenting soil indigenous fungi may be more effective in comparison with a conventional biostimulation approach. These improved biodegradation results obtained by using multiple indigenous microorganisms for bioaugmentation agrees with previous similar studies (Atagana et al., 2006; Mancera-López et al., 2008).

**FIGURE 2:**
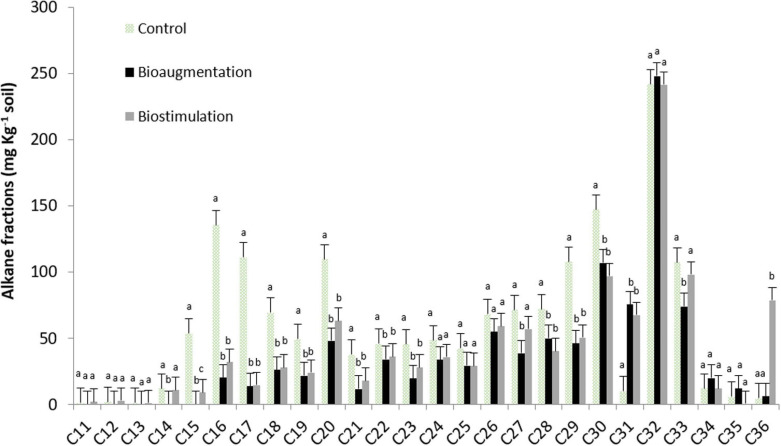
Alkane fractions in control soil and treatments after 120 days of incubation in microcosms experiments. Same lowercase letters indicate, between microcosms and vs. control soil, respectively, lack of statistically significant difference (*P* < 0.05).

The biodegradation patterns of alkane fractions in microcosms B and BS were markedly different throughout time, compared to C. Both treatments (B and BS) displayed complete biodegradation of the light aliphatic fractions, up to C16, and an intense biodegradation of aliphatics up to C28–C30, while the higher molecular weight fractions (C30–C36 range) remained largely undegraded (Figure 2). The chromatographic profile of the soil (Supplementary Figure 2) shows the absence of resolved alkanes, while only pristane and phytane were identified initially in the saturated fraction as the main resolved isoprenoids, which were fully degraded in both treatments after 120 days (B and BS).

#### Polycyclic Aromatic Hydrocarbons

**FIGURE 3:**
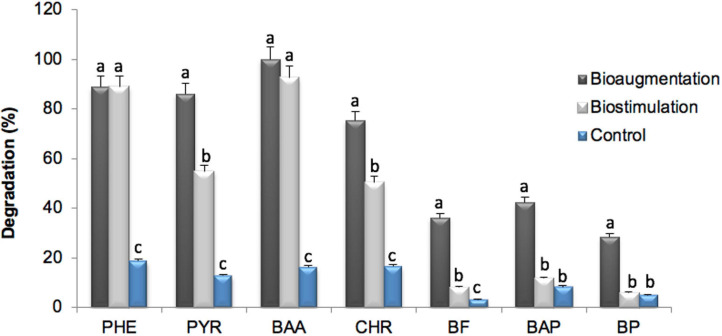
Degradation of PAHs (%) after 120 incubation days in experimental conditions. (Mean values ± SD for three replicates). B, Bioaugmentation; BS, Biostimulation; PHE, (phenanthrene, three rings PAH); PYR, (pyrene, four rings PAH); BAA, [benzo(a) anthracene, four rings PAH]; CHR, (Chrysene, four rings PAH); BF, [benzo(b,k)fluoranthene, five rings PAH]; BAP, [benzo(a)pyrene, five rings PAH]; BP, [benzo(g,h,i)perylene, six rings PAH]. Different lower-case letters within treatments indicate the occurrence of significant differences between them (*P* < 0.05). Anthracene was at very low values, below the detection limit (i.e., 2 mg Kg^– 1^), reason why it has not been included in the figure.

Biodegradation percentages were calculated in relation to the PAH contents of the control soil (C) after 120 days, in order to consider only the effects of soil treatments (B and BS) (Supplementary Table 2). An increase of total PAHs biodegradation compared to C was observed in B and BS microcosms, though biodegradation patterns were different depending on the number of aromatic rings of PAHs (Figure 3). In general terms, B displayed a significantly improved removal (*P* < 0.05) of the analyzed 16 PAHs, compared with BS (74.04 ± 1.15% vs. 48.44 ± 0.61% biodegradation respectively). Bioaugmentation with autochthonous selected fungi, mainly of the phylum Ascomycota, may thus play an important role in the bioremediation of PAHs.

Regarding 3- and 4-ringed PAHs (phenanthrene, pyrene, benzo(a)anthracene and chrysene), fungal bioaugmentation led to a final average degradation efficiency of 87.56%, which was significantly higher than that of 71.51% with biostimulation (*P* < 0.05). The removal of benzo(a)anthracene was complete in both treatments but phenanthrene remained at a final concentration of 1 mg Kg^–1^. In the particular case of pyrene and chrysene, the highest biodegradation levels were observed in B, with 86.03% and 75.4%, in contrast to the 54.66 and 50.26% observed in BS.

Recalcitrant 5- and 6-ringed PAHs [benzo(b,k)fluoranthene, benzo(a)pyrene, benzo(g,h,i)perylene] were biodegraded in average to a substantially greater extent in B than in BS (53.33 and 7.91% respectively; *P* < 0.05). The detailed PAHs biodegradation efficiencies in B and BS were respectively: 36.12 and 8.17% for benzo(b,k)fluoranthene; 42.27 and 11.73% for benzo(a)pyrene, and 28.27 and 5.88% for benzo(g,h,i)perylene. This result is rather coincident with the criterion that correlates the increase in recalcitrance of PAHs with the molecular weight and number of aromatic rings, as described previously (Atagana, 2009; Serrano Silva et al., 2009), though such conclusions have been contested by other authors (Tiehm et al., 1997; Rafin et al., 2000; Wu et al., 2008; Hesham et al., 2016; Fayeulle et al., 2019). The efficient removal of benzo(a)pyrene could be explained by the relatively high initial concentration of this PAH, which could generate concentration gradients that enhanced its mass transfer rate toward microorganisms, as described previously by Li et al. (2007).

As of the 3- and 4-ringed PAHs biodegradation in BS and B microcosms, the remaining concentrations comply with the targets from the European Union and the Argentinian legislation (Table 1) for soils dedicated to industrial and agricultural uses. However, in the BS treatment, the 5–6 ringed PAHs [benzo(b,k)fluoranthene, benzo(a)pyrene and benzo(g,h,i)perylene] were still above the threshold values for agricultural and residential soils, while in B only benzo(g,h,i)perylene remained above regulatory threshold values and might need prolonged bioaugmentation.

Our results generally agree with similar experiments comparing bioaugmentation of indigenous filamentous (non-ligninolytic) fungi vs. biostimulation with nutrients and water on soils heavily polluted with PAHs, performed by Wu et al. (2008) and Mancera-López et al. (2008). These studies confirm that the use of filamentous fungi isolated from PAH-contaminated soil presents the advantage that they are adapted not only to the presence of contaminants but also to the specific environmental conditions of the site. D’Annibale et al. (2006) showed that fungal bioaugmentation in a contaminated soil with specific ascomycetes from the genera *Allescheriella*, *Stachybotrys*, and *Phlebia* resulted in a significant PAH removal and soil detoxification. However, these results are not consistent with the findings of Serrano Silva et al. (2009), who used individual fungi and bacterial as well as a fungal consortium, and concluded that bioaugmentation did not significantly affect the biodegradation efficiency for naphthalene, phenanthrene, anthracene, pyrene, benzo(a)anthracene, and benzo(a)pyrene, except for *Aspergillus*, compared to biostimulation. An alternative bioaugmentation approach for the removal of HMW-PAHs has been reported by Boonchan et al. (2000) and Li et al. (2007) with a significantly improved degradation of anthracene, fluoranthene, pyrene, chrysene, benzo(a)anthracene, benzo(a)pyrene, and dibenz(a,h)anthracene with a combined fungal-bacterial consortium that mineralized these compounds as sole carbon and energy source.

Many strains that are capable of efficient degradation of PAHs under laboratory conditions exhibit a reduced capacity when applied in aged polluted soils for 5- and 6-ringed PAHs removal. This is not only due to the complex chemical structures of 5–6-ring PAHs and their low water solubility and high stability, but also because of limited adaptability of the isolated strains to the soil environment and the competition of soil indigenous microbial populations (Zhao et al., 2017). In B microcosms, the inoculation of autochthonous fungi associated to the genera *Penicillium, Ulocladium, Aspergillus*, and *Fusarium* improved significantly the biodegradation of the 5-ringed PAHs BF, BAP, and BP, probably because of the good adaptability of the reintroduced strains. These results are coincident with other studies focused on BAP degradation by non-ligninolytic fungi (Boonchan et al., 2000; Rafin et al., 2000; Veignie et al., 2002; Fayeulle et al., 2014, 2019). Fayeulle et al. (2014) demonstrated that CYP is one of the enzymes involved in BAP mineralization by *F. solani*.

It is noteworthy that the range of TPH biodegradation observed (EPA 418) in B and BS corresponded to the decrease of total C observed (13 and 6% reduction), from initial (12.70% of soil in the polluted soil to 11.10 and 11.96% of total C respectively) (Supplementary Table 1). This decrease of total C would reveal that a high mineralization of TPH, which represents 35% of total C in the polluted soil, occurred in both treatments. Besides TPH, among the other pollutant fractions are the polar fraction (containing photooxidation products, heterocyclic compounds and partial and end products from microbial metabolism), as well as the resins and asphaltenes. This fact indicates that most of the observed biodegradation could be linked to the complete mineralization of hydrocarbons, minimizing the accumulation of polar metabolites. In this regard, bioaugmentation was associated to a significant increase in hydrocarbon biodegradation (*P* < 0.05) and to a decrease of ecotoxicity (*P* < 0.05), when compared to biostimulation and the untreated control soil. Such results point to the fact that the initial partial oxidation promoted by the fungal enzymes from the inoculum could act synergically with the activity of the native soil microbiota for the subsequent mineralization of both parental PAHs and TPHs, and the generated partially oxidized and more bioavailable metabolites. The increased fungal activity through bioaugmenting native fungi could overcome the metabolic limitations from bacteria, particularly in what concerns the initial oxidation of hydrocarbons, which was especially evident to enhance biodegradation of the parental 5- and 6-ringed PAHs by cometabolism as recently described (Sun et al., 2020). On the other hand, bioaugmentation using native soil organisms avoids the issue of altering the biodiversity of ecosystems with the introduction of exogenous species, fulfilling the Nagoya Protocol principles (Smith et al., 2017). Although the main problem of invasive species concerns primarily the macrobiota, it is worth highlighting the benefits of using the native microflora for the restoration of the soil matrix.

### Effects on Soil Ecotoxicity

**FIGURE 4:**
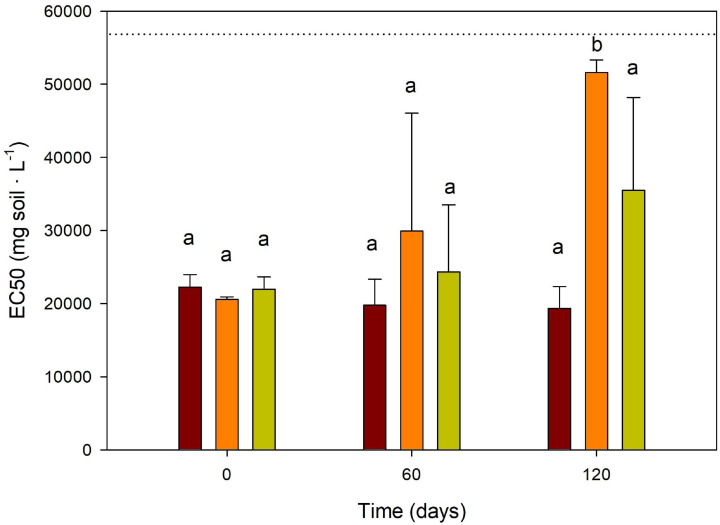
Microtox ecotoxicity solid phase assays of soil (EC50 values expressed as mg soil L^–1^) in the different treatments over 120 days of incubation. Dotted line represents EC value of clean soil (56,682 mg L^–1^) collected in the vicinity of polluted site. Brown bar: contaminated control soil; Orange Bar Bioaugmentation; Green Bar (Biostimulation). Different lower-case letters within treatments indicate the occurrence of significant differences between them (*P* < 0.05).

After 60 days of incubation, no significant differences were observed in terms of ecotoxicity between the different soil treatments. However, after 120 days, a remarkable reduction of ecotoxicity (increase of the EC50 value) was observed with fungal bioaugmentation (B), compared to biostimulation (BS) and the control (C) untreated soil (*P* < 0.05, Figure 4), so that low toxicity values comparable to the clean soil were obtained (Figure 4). TPH biodegradation efficiencies of 34–40% obtained with B also resulted in a significant decrease in toxicity. This phenomenon could be explained by a decrease of the content of secondary metabolites, due to higher mineralization rates of hydrocarbons during biodegradation, thanks to the synergistic interactions between the autochthonous soil microbiota and the bioaugmented fungi. The implemented *V. fischeri* bioluminescence assay has been reported as the most sensitive ecotoxicological test for a wide range of environmental contaminants, compared to other bacterial assays such as nitrification inhibition, respirometry, ATP luminescence and enzyme inhibition (Cotou et al., 2002). It has also shown good correlations with other standard acute toxicity assays across a wide spectrum of toxicants in soils and sediments (El-Alawi et al., 2002; Abbondanzi et al., 2003). Although mostly applied for aqueous phase samples and organic extracts, the test can also be conducted directly on soil and sediment samples to measure the toxicity due to the bioavailable fraction (Parvez et al., 2006). Nevertheless, the availability of PAHs due to the extractive saline solution of the method rather than their total concentration may affect the results, as reported by Harkey and Young (2000). In our case, given the rather low quantitative contribution of PAHs to TPHs [88.17 mg kg^–1^ of PAHs on 45,000 mg kg^–1^ of TPH (IR) or 16,114 mg kg^–1^ TPH C10-C35], the polar metabolites or partial oxidation products affecting the toxicity test could derive mainly from the hydrocarbons comprised in TPHs and not only from the HMW-PAHs. This justifies further the use of TPHs for the statistical regression analysis and Microtox readings. In this sense, the different degradation of these compounds described previously between B and BS is also coherent with the results of this test.

### Hydrocarbon-Degrading Microbial Populations

Bioaugmentation caused an increase of two to three orders of magnitude of the total bacterial heterotrophic population counts, compared to those from the initial soil, up to values of 2.54 × 10^8^ MPN g soil^–1^ and 1.84 × 10^9^ MPN g soil^–1^ after 30 and 60 days of incubation, respectively (Table 3). In addition, from day 60–120, the alkane-degrading bacterial populations increased by one order of magnitude and stabilized at 7.8 × 10^7^ MPN g soil^–1^. Similarly, PAHs-degrading bacteria increased one and three orders of magnitude at days 30 and 60, respectively, but decreased down to a basal level of 7.1 × 10^4^ MPN g soil^–1^ after 120 days.

**TABLE 3:** Time-course evolution of the most probable number (MPN) of specific microbial populations during microcosm incubations.

Treatment	Process days	Heterotrophs (MPN ml^–1^)^a^	Aliphatic degraders (MPN g soil^–1^)^a^	PAHs degraders (MPN g soil^−1^)^a^
Soil	0	1.26 ± 0.17 × 10^6^ aA	1.50 ± 0.18 × 10^4^ aA	1.04 ± 0.19 × 10^6^ Aa
	30	1.30 ± 0.18 × 10^6^ aA	1.05 ± 0.18 × 10^6^ bA	1.50 ± 0.18 × 10^4^ bA
	60	6.30 ± 0.18 × 10^5^ bA	8.30 ± 0.19 × 10^4^ aA	6.90 ± 0.19 × 10^4^ bA
	120	1.20 ± 0.17 × 10^5^ bA	7.00 ± 0.17 × 10^3^ cA	6.30 ± 0.18 × 10^4^ bA
Bioaugmentation	30	2.54 ± 0.19 × 10^8^ aB	1.50 ± 0.18 × 10^4^ aB	1.50 ± 0.18 × 10^7^ aB
	60	1.84 ± 0.19 × 10^9^ bB	4.70 ± 0.19 × 10^5^ bB	5.16 ± 0.18 × 10^7^ aB
	120	2.9 ± 0.20 × 10^8^ aB	7,80 ± 0,41 × 10^7^ cB	7.10 ± 0.19 × 10^4^ bA
Biostimulation	30	7.20 ± 0.17 × 10^7^ aB	1.40 ± 0.17 × 10^6^ aB	1.20 ± 0.17 × 10^5^ aA
	60	6.30 ± 0.18 × 10^5^ bA	1.10 ± 0.17 × 10^6^ aB	1.80 ± 0.18 × 10^4^ aA
	120	6.00 ± 0.19 × 10^5^ bA	1.20 ± 0.18 × 10^5^ bB	1.30 ± 0.19 × 10^4^ aA

*^a^Means and standard deviations of three replicates; soil dry weight basis.*

*Same lowercase and uppercase letters indicate, within every microcosm, lack of statistically significant difference (*P* < *0.05*) for every microbial population along time and vs. control soil, respectively.*

In the biostimulation treatment, the aliphatic-degrading bacterial populations increased in numbers by two orders of magnitude, up to 1.4 × 10^6^ MPN g soil^–1^, while the total heterotrophs initially decreased by one order of magnitude with respect to the control soil without treatment, remaining stable until day 120, at 6 × 10^5^ MPN g soil^–1^. The addition of water and nutrients did not cause a biostimulation of the PAHs-degrading bacterial populations, which remained stable in the order of 10^4^ MPN g soil^–1^ throughout the study time. Interestingly, during fungal biaugmentation the aliphatic-degrading bacteria achieved the highest counts in the late stages (60–120 days), with values above 10^7^ MPN × g^–1^ (2.5 magnitude order higher than those observed in the BS treatment). Such high bacterial counts were also coincident with the maximum biodegradation of TPH of late stages in B (Figure 1). These results demonstrate that the inoculation of the autochthonous fungi promoted the establishment of active hydrocarbon-degrading bacterial populations that biodegraded both aliphatic hydrocarbons (late stages) and PAHs (early and late stages) in aged-polluted soils. Similarly, D’Annibale et al. (2006) also described an increase of hydrocarbon-degrading bacterial populations after fungal bioaugmentation, which could be linked to a potential increase of PAHs bioavailability due to the generation of more polar metabolites by hydrocarbon-degrading fungi (Meulenberg et al., 1997; Capotorti et al., 2005).

#### Effects of Fungal Bioaugmentation on Bacterial Biodiversity

**FIGURE 5:**
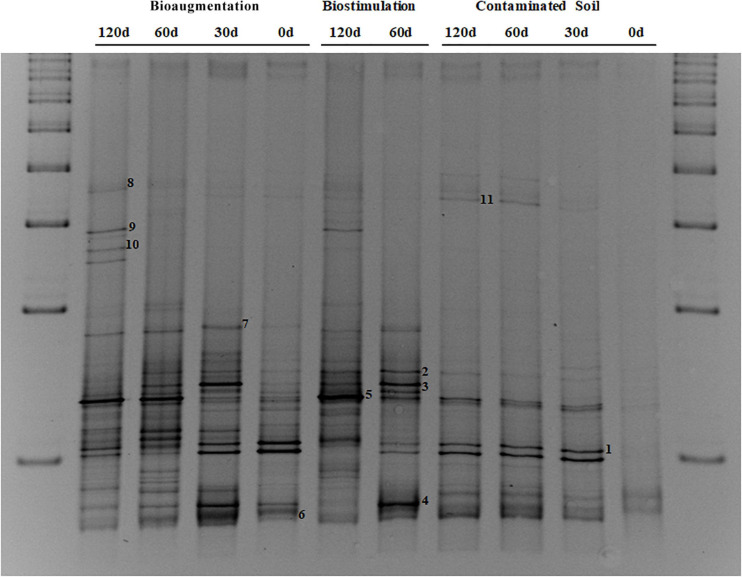
DGGE profiles of bacterial 16S rDNA from microcosmos assays at 0, 30, 60, and 120 days of incubation. A standard ladder (L) has been added at both gel ends in order to check the DNA migration homogeneity. Bacterial bands have been named from B1 to B11.

The DGGE profile of bacterial ribotypes shows that the original hydrocarbon-contaminated soil (C) had a stable community of bacteria, with a relatively low number of predominant bands, which evolved during the biodegradation of aromatic and saturated hydrocarbons toward a more complex microbial community structure (Figure 5). This relatively low initial biodiversity may be explained because, despite recent landfarming practices, this soil was exposed to relatively high contamination levels and extreme climatic conditions for several years. It must also be highlighted that with the DGGE technique only the predominant organisms, which usually account for more than 1% of the total relative abundance, can be depicted. For this reason, in highly diverse environments such soil samples, discrete fingerprint bands may not always be discernible, leading to smearing or poorly resolved patterns (Green et al., 2010). Such unresolved DGGE patterns were observed in this work for the fungi (results not shown), but the long-term effects of fungal bioaugmentation were seen on the bacterial population shifts, indicating that at least some strains of the re-introduced fungi survived the microcosm experimental conditions.

Four relevant DGGE bands present in the control soil samples were successfully sequenced and phylogenetically assigned (Table 4). The sequence from the intense band 1 was identical to the type strain of *Bacillus thioparans* and, though less dominant than in the control soil, was still visible in most biostimulated and bioaugmented microcosm samples. This thiosulfate-oxidizing bacterium has previously been found to also degrade and grow with crude oil as its sole carbon and energy source (Syakti et al., 2019). Another dominant ribotype from the control soil was distantly related to *Immundisolibacter cernigliae* as the closest known species (band 5, 90% sequence homology), but it was identical to an uncultured bacterium in the phylum *Proteobacteria* from a pilot-scale bioremediation process of a hydrocarbon-contaminated soil (Militon et al., 2010). It might thus correspond to an undescribed *Proteobacterium* that occupies environmental niches that are rich in petroleum hydrocarbons. Two additional ribotypes were detected with lower amounts in the control soil, but the band intensity increased upon biostimulation. Band 2 was highly homologous to a number of species from the genus *Sphingobium* (98.60%) and was also practically identical to the sequence of an uncultured bacterium obtained from a PAHs contaminated soil. Similarly, the sequence from band 11 somewhat matched those from type strains in the *Fermentimonas* (95.28%) and *Lascolabacillus* genera, but it was highly similar to an uncultured bacterium from a PAHs contaminated soil.

**TABLE 4:** Identification of excised and amplified DGGE bands (Figure 5) through nucleotide sequence BLAST to the GenBank database.

Band	Accession number	Detection^*a*^			Closest match in GenBank (accession no.) Closest type strain in GenBank (accession no.)	Similarity^*b*^ (%)	Classification (Phylum/Order)
				
		CS	BS	BA			
1	JF729186	+	+	+	*Bacillus thioparans* strain BMP-1 (NR_043762)	100.00	*Firmicutes/Bacillales*
2	JF729187	+	+	+	Uncultured bacterium clone SPN0-300day-48 (MF085114) *Sphingobium aquiterrae* SKLS-A10 (MF980915)^*T*^ *Sphingobium cloacae* JCM 10874 (AP017655)^*T*^ *Sphingobium phenoxybenzoativorans* SC_3 (NR_135895)^*T*^ *Sphingobium baderi* LL03 (NR_118315)^*T*^ *Sphingobium wenxiniae* JZ-1 (NR_116773)^*T*^ *Sphingobium faniae* strain JZ-2 (FJ373058)^*T*^	99.53 98.60	*Proteobacteria/Sphingomonadales*
3	JF729188	–	+	+	*Promicromonospora viridis* NEAU-JGR1 (NR_164914)^*T*^ *Promicromonospora thailandica* S7F-02 (NR_113177)^*T*^	99.77	*Actinobacteria/Micrococcales*
4	JF729189	–	+	+	*Olivibacter* sp. DB4 (MG571618) *Olivibacter ginsenosidimutans* BS18 (JQ349042)^*T*^	98.04 94.52	*Proteobacteria/Sphingomonadales*
5	JF729190	+	+	+	Uncultured bacterium clone AMOH12 (AM935143) *Immundisolibacter cernigliae* TR3.2 (NR_156801)^*T*^	100.00 90.35	*Proteobacteria/Immundisolibacterales*
6	JF729191	–	–	+	Uncultured bacterium clone 16S-T6-1-H5 (KC664088) *Streptacidiphilus bronchialis* DSM 106435 (CP031264)^*T*^ *Streptomyces barkulensis* RC 1831 (NR_133869)^*T*^ *Streptomyces griseoplanus* NRRL-ISP 5009 (NR_118417)^*T*^ *Streptomyces atacamensis* C60 (NR_108859)^*T*^ *Streptomyces qinglanensis* 172205 (NR_109303)^*T*^ *Streptomyces fenghuangensis* GIMN4.003 (NR_117502)^*T*^ *Streptomyces nanhaiensis* SCSIO 01248 (NR_108633)^*T*^ *Streptomyces radiopugnans* R97 (NR_044013)^*T*^	96.52 96.23	*Actinobacteria/Streptomycetales*
7	JF729192	–	+	+	*Sphingopyxis macrogoltabida* NBRC 15033 (NR_113720)^*T*^	100.00	*Proteobacteria/Sphingomonadales*
8	JF729193	–	–	+	Uncultured bacterium clone SPN2000-90day-62 (MF314694) *Fermentimonas caenicola* ING2-E5B (NR_148809)^*T*^ *Lascolabacillus massiliensis* SIT8 (NR_144720)^*T*^	99.79 95.28	*Bacteroidetes/Bacteroidales*
9	JF729194	–	+	+	Uncultured bacterium clone 4-54 (KC521985) *Ohtaekwangia koreensis* (NR_117435)^*T*^	99.52 93.79	*Bacteroidetes/Cytophagales*
10	JF729195	–	–	+	Uncultured bacterium clone CMJG7 (AM935906) *Azoarcus olearius* DQS4 (CP016210)^*T*^	99.73 90.72	*Proteobacteria/Rhodocyclales*
11	JF729196	+	+	+	Uncultured bacterium clone (JX310909) *Fermentimonas caenicola* ING2-E5B (NR_148809)^*T*^ *Lascolabacillus massiliensis* SIT8 (NR_144720)^*T*^	100 96.42	*Bacteroidetes/Bacteroidales*

*^T^Type strain.*

*^a^Band detection (+) above 1% of relative intensity in the control soil (CS); biostimulated microcosms (BS) and bioaugmented microcosms (BA).*

*^b^Sequences were matched with the closest relative from the GenBank database.*

As for the biostimulated and bioaugmented microcosms, the microbial communities displayed a more dynamic pattern, which progressively evolved from the original control soil. The ribotype sequences from bands 3 and 4 became particularly intense after 60 days of biostimulation. The sequence of the band 3 was practically identical to those from two type strains of the genus *Promicromonospora*, while the second was closely related to *Olivibacter*. Not surprisingly, species from these genera have previously been described to be enriched in environments polluted with oil hydrocarbons (Szabó et al., 2011; Wu et al., 2017). Band 7 was also present in both biostimulated and bioaugmented microcosms and corresponds to *Sphingopyxis macrogoltabida*. This species is closely related to the previously mentioned *Sphingobium* and other related *Sphingomonads* in that they have commonly been described in association with petroleum hydrocarbons (Kertesz and Kawasaki, 2010). Interestingly, band 6 appeared to be specific from the soil bioaugmented with fungi and was somewhat related to a number of species in the genus *Streptomycetes* and *Streptacidiphilus*. Members from these taxa display a filamentous growth and are well-known as secondary metabolite producers in response to syntrophic/antagonistic interactions with fungi (Jones et al., 2017). As with the previous, bands 8 (Bacteroidales), 9 (Cytophagales), and 10 (Rhodocyclales) they all share the fact that they were enriched during fungal bioaugmentation, but their sequences are closely related to yet undescribed bacteria that nevertheless have been found in different hydrocarbon polluted environments.

**FIGURE 6:**
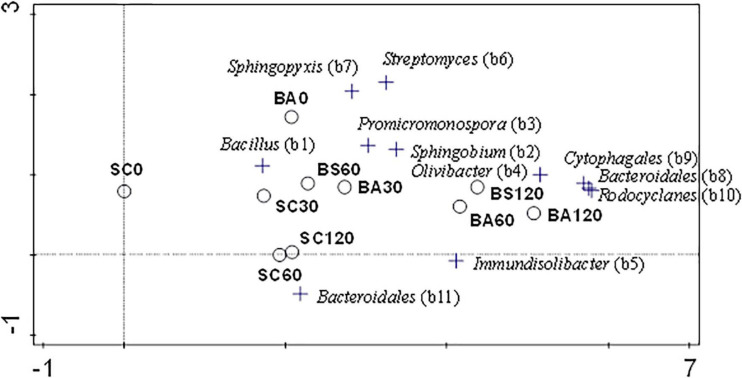
Detrended correspondence analysis (DCA) on the relative DGGE band intensity of bacterial ribotypes from microcosm samples taken at different time and with treatments, as detected from the DGGE profiles (Figure 5). Only those bands with a relative band intensity higher than 1% were considered.

A gradient analysis was performed on the bacterial community DGGE profiles from Figure 6 by multivariate detrended correspondence analysis (DCA). A “species relative abundance” matrix of 47 ribotypes was generated from the bands’ positions and relative intensity. Sample and sequenced ribotype scores were represented in a biplot that encompassed a 36% of the explained variation and the first axis represented a beta diversity (gradient length) of 5.08 (Figure 6 and Supplementary Table 3). In general, sample scores were chronologically arranged through the main axis, thus showing that incubation time affected the microbial community dynamics. This effect was evident with biostimulation but it was particularly marked with fungal bioaugmentation, in that bacterial profiles from samples taken at equal times tended to be more differentiated compared to those from the control soil. The DCA biplot also depicts the enrichment of bands 8 (*Bacteroidales*), 9 (*Cytophagales*) and 10 (*Rhodocyclales*) in bacterial populations upon fungal bioaugmentation.

Bioaugmentation is often considered to tackle bioremediation of the most recalcitrant compounds (Atlas and Philip, 2005), and under extreme environmental conditions. In the present study, bioaugmentation with autochthonous fungi may facilitate the establishment of fungal and bacterial consortia responsible for a more efficient oxidation and complete biodegradation of both TPH and HMW-PAHs. A similar strategy has been proposed previously by Boonchan et al. (2000) and Sasek et al. (1998). Even when the culturable isolated strains may not be representatives of the dominant or active fungal diversity of the soil, the active fungal biomass introduced with bioaugmentation prompted changes in bacterial and fungal soil population, resulting in the selective and sustained growth and biodegradation activity that explains the success of the experimental approach taken in this study.

## Conclusion

Bioaugmentation of indigenous fungi in an aged oil-polluted soil caused an important shift of the bacterial populations, which was also linked to a significant increase of the TPH (C_10_–C_35_) and HMW-PAHs biodegradation efficiency, when compared to a standard biostimulation alternative strategy. Biodegradation of PAHs has commonly been associated to the ligninolytic system of the WRF. However, pilot and full-scale projects investigating the bioremediation of TPHs and PAHs after soil bioaugmentation with white-rot fungi (WRF) have often yielded unconvincing results. Bottlenecks in these bioremediation processes included the inability of the WRF to compete with the native soil microbes, along with specific nutritional requirements of lignocellulosic materials. In addition, the metabolism of hydrocarbons by WRF is also related with the generation of toxic metabolites, which might create an elevated ecotoxicity problem.

In this microcosm study we demonstrated that bioaugmentation of autochtonous fungal species might enhance the bioremediation of aged hydrocarbon pollution and overcome the vastly described inconveniences of white-rot fungi’s use for the remediation of aged-industrially-polluted soils enriched in heavy hydrocarbons. Cytochrome P450 and fungal laccases may play a very relevant role in the fungal bioremediation of oil hydrocarbons, without increasing the ecotoxicological risk. Furthermore, the fact that the selected fungi were rather fast growing and cosmopolitan species might facilitate the isolation, scaling-up and feasibility of bioaugmentation at field conditions, overcoming the previously mentioned limitations of using WRF. This study also corroborated the hydrocarbonoclastic potential of using *Fusarium*, *Aspergillus* and *Penicillium* strains under soil conditions. Previous studies in the laboratory have shown that species from these genera, particularly *F. solani*, were able to assimilate benzo[a]pyrene as the sole source of carbon and energy. Melanized fungi, such as the bioaugmented *Ulocladium* sp., have also been associated to hydrocarbon biodegradation due to the oxidative enzymatic machinery required for the biosynthesis of melanin (laccases, phenol oxidases, etc.).

Besides the direct action of fungi on biodegradation, molecular profiling by DGGE also demonstrated that fungal bioaugmentation with indigenous fungi exerted an important influence on the soil bacterial populations toward a more diverse and synergistic microbial community. Enriched hydrocarbon-degrading bacteria during fungal bioaugmentation were associated to a number of undescribed species in the orders Cytophagales, Bacteroidales, and Rhodocyclales, which have nevertheless been previously detected in similar hydrocarbon polluted sites through molecular means. This finding provides a first insight on the dimension of the yet unknown biodiversity and interactions between hydrocarbonoclastic microbial communities, which deserves further fundamental and applied research. The further integration of selective microbial isolation and screening methods with recent advances on high throughput DNA/RNA-seq approaches will allow for a detailed description of hydrocarbonoclastic fungi and bacteria from polluted soils, in terms of their taxonomy, ecophysiology, and function. Such information will be the key for the design of optimized and minimally invasive bioremediation techniques.

## Data Availability Statement

The datasets presented in this study can be found in online repositories. The names of the repository/repositories and accession number(s) can be found in the article/Supplementary Material.

## Author Contributions

MM and MV conceived the experimental design. MM collected soil samples, isolated the fungal strains, and wrote the original draft. MM and MG performed the microcosm’s experiments and processed the samples in the laboratory. MG, FP-B, XM-V, and MV helped analyzing and interpreting data. MM, MG, FP-B, XM-V, and MV wrote and revised the manuscript. All authors contributed to read and approved the final version.

## Conflict of Interest

The authors declare that the research was conducted in the absence of any commercial or financial relationships that could be construed as a potential conflict of interest.
