# Supplementary material for: Bioaugmentation of Native Fungi, an Efficient Strategy for the Bioremediation of an Aged Industrially Polluted Soil With Heavy Hydrocarbons
Source: Front Microbiol. 2021 Mar 31;12:626436. doi: 10.3389/fmicb.2021.626436 (PMC8044458; doi:10.3389/fmicb.2021.626436)
Supplement: Supplementary file 1 [file Data_Sheet_1.pdf]

Supplementary material:

Tables:

Table S1. Elemental analysis of CHNS

Soil	C (%)[a]	H (%)[a]	N (%)[a]	S (%)[a]
**Clean soil**	2.18±0.01 a	1.99±0.01 a	0.04±0.01 a	0.15±0.03 a
**Control soil (C)**	12.70±0.86 bA	2.87±0.01 bA	0.17±0.04 bA	0.63±0.02 bA
**Bioaugmentated soil (B) 120 d**	11.10±0.17 bB	2.62±0.02 bB	0.22±0.01 bA	0.56±0.00 bA
**Biostimulated soil (BS) 120d**	11.96±0.04 bC	2.70±0.02 bA	0.18±0.01 bA	0.61±0.03 bA

[a] Data are means ± standard deviations of three replicates.
Same lowercase and uppercase letters indicate lack of statistically significant difference ($P< 0.05$) between pristine soil and microcosms and between control contaminated soil with microcosms (Biostimulation and Bioaugmentation treatments), respectively.

Table S2. Total biodegradation of polycyclic aromatic hydrocarbons (PAHs)

PAHs degradation (%)			
**Abbreviation**	**Nº of rings**	**B**	**BS**
PHE	3	88.88 ± 0.06 a	88.88 ± 0.83 a
PYR	4	86.03 ± 4.74 a	54.66 ± 0.07 b
BAA	4	100 ± 0.38 a	92.65 ± 0.52 a
CHR	4	75.34 ± 0.45 a	50.26 ± 0.46 b
BF	5	36.12 ± 2.31 a	8.17 ± 1.11 b
BAP	5	42.27 ± 2.68 a	11.73 ± 0.13 b
BP	6	28.27 ± 1.51 a	5.88 ± 0.45 b
Average degradation 3,4-rings		87.56	71.61
Average degradation 5,6-rings		53.33	7.91
Average total PAHs degradation		74.05	48.44

B: Bioaugmentation, BS: Biostimulation

PHE: Phenanthrene, PYR: Pyrene, BAA: Benz(a) anthracene, CHR: Chrysene, BF Benz(b,k)fluoranthene, BAP: Benz (a)pyrene, BP: Benz(g,h,i)perylene

Equal lowercase letters indicate lack of statistically significant differences ($P < 0.05$) for every PAH between biostimulation and bioaugmentation treatments after 120 days of trial.

Table S3. Summary of the statistical parameters from the detrended correspondence analysis (DCA) on the bacterial DGGE profiles (Figure 5).

**Parameter**	**Axis 1**	**Axis 2**	**Axis 3**	**Axis 4**
Eigenvalues	0.712	0.19	0.06	0.02
Explained variation (cumulative)	28.37	36.10	38.36	39.09
Gradient length	5.08	1.72	1.70	0.99
Total variance	2.51223			

Figures:

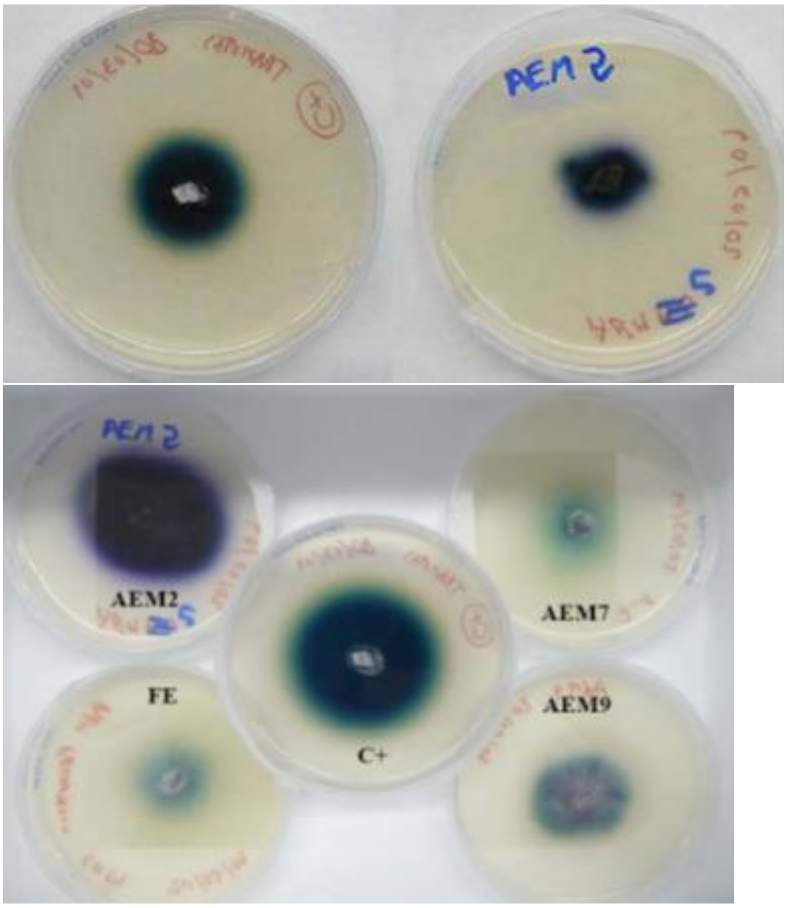

Figure S1. Enzyme activity tests on isolated strains.

A) Laccase activity. Isolated positive strains and Positive Control (center) in ABTS Mineral Medium Agar. B) Polyphenol oxidase activity. Left: Positive control *Trametes versicolor*; Right: Isolated strains AEM2, *Ulocladium sp.* in Malt Extract Agar.

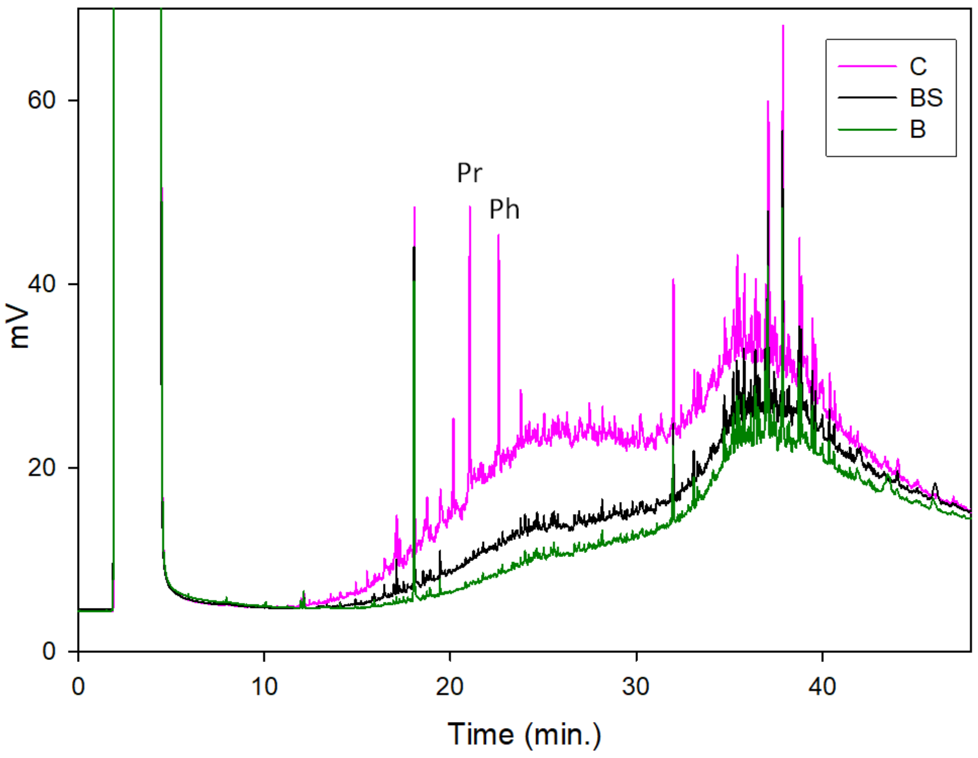

Figure S2. Soil chromatogram profiles (GC-FID) of microcosms of contaminated soil (C), biostimulated soil (BS) and bioaugmented soil (B), after 120 days of incubation.

Pr: Pristane ; Ph: Phytane.
